# Waitlist mortality risk (US-CRS score) distribution among status 1 and 2 adult heart transplant candidates with exceptions

**DOI:** 10.1016/j.jhlto.2026.100706

**Published:** 2026-10-12

**Authors:** Najiya Fatma, Suhani S. Patel, Grace R. Lyden, Nader Moazami, Brian Wayda, William F. Parker, Sommer E. Gentry

**Affiliations:** a Department of Surgery, NYU Grossman School of Medicine, New York, New York; b Department of Medicine, University of Minnesota, Minneapolis, Minnesota; c Department of Cardiothoracic Surgery, NYU Grossman School of Medicine, New York, New York; d Department of Medicine, University of Chicago, Chicago, Illinois

**Keywords:** waitlist mortality, continuous risk score, adult heart transplantation, exceptions, status 1 and status 2 candidates

## Abstract

**Background:**

The US-candidate risk score (US-CRS) predicts waitlist mortality among heart transplant candidates using 7 laboratory, cardiovascular, and cardiac device usage variables on a 50-point scale, with higher scores indicating greater mortality risk. We assessed whether US-CRS accurately assigns mortality risk for exception candidates, and whether the alignment of allocation priority with waitlist mortality risk (US-CRS) differs between candidates with and without exceptions.

**Methods:**

We compared the performance of US-CRS in candidates with and without exceptions using receiver operating curves. We compared US-CRS distributions using the Kolmogorov-Smirnov (KS) test and examined the proportion of candidates below specific score thresholds in each group. We also compared candidates with and without exceptions using Wilcoxon Rank-Sum test for continuous variables and Pearson’s Chi-squared test or Fisher exact test for categorical variables.

**Results:**

US-CRS is accurate for measuring mortality risk among exception candidates as it is among non-exception candidates. Patients with higher waitlist status had higher median US-CRS. However, the median US-CRS of Status 1 candidates without exceptions (50 [IQR 48–50]) was significantly higher than for candidates with exceptions (45 [36–48]; [p≤0.05]), and similarly for Status 2 (46 [41–49] vs. 42 [32–47]; [p≤0.05]). Notably, 33.13% of Status 1 and 41.28% of Status 2 candidates with exceptions had US-CRS below 40, compared to only 1.84% and 19.20% of candidates without exceptions, respectively. Conversely, among candidates in Status 3 and 4, candidates with exceptions generally had higher US-CRS than candidates without exceptions.

**Conclusions:**

Allocation priority under the status system does not align with estimated mortality risk; misalignment is greater for candidates with exceptions. While some exception candidates in Status 1 and 2 do have high estimated mortality risk and legitimately need exceptions, many of these exceptions appear to have been granted to candidates with relatively low medical urgency.

## Background

The current adult heart allocation system primarily considers hemodynamic criteria and levels of mechanical and inotropic support to categorize candidates in 6 statuses that reflect their medical urgency for transplant.^1^ Alternatively, physicians can apply for exceptions for candidates to reach a higher status if they believe the candidate has medical urgency comparable to standard-criteria listed candidates, hereafter referred to as candidates without exceptions. Exception requests are reviewed by the Regional Review Board (RRB) and must be renewed every 14 days after approval.^1^ Between October 2018 and September 2024, among 26,300 candidates, 38.6% used exception requests, with a statistically significant increase over time, particularly in high priority statuses.^2^, ^3^ Denials for exception requests are very rare (<5%).^4^

This growing reliance on exceptions in reaching higher statuses raises concerns about fairness and clinical necessity, as candidates with exceptions may have lower waitlist mortality risk than candidates without exceptions.^5^ Existing studies comparing observed waitlist mortality between candidates with exceptions and without exceptions have shown varied results; some studies reported higher transplant rates for candidates with exceptions without increased risk of observed waitlist mortality,^5^ while others found significantly lower observed waitlist mortality for candidates with exceptions in higher statuses (Status 1 and 2), or mortality rates comparable to candidates without exception.^6^, ^7^, ^8^ The US-candidate risk score (US-CRS) system estimates predicted waitlist mortality using 7 clinical variables (laboratory, cardiovascular, cardiac device usage) among heart transplant candidates in a 50-point scale, with higher scores indicating greater mortality risk.^9^ Previous studies have concluded that candidates with exceptions have lower average waitlist mortality than candidates without exceptions. However, candidates with exceptions are likely a heterogenous group in terms of risk, and the US-CRS score might be capable of capturing this variation and prioritizing candidates accordingly.

In this study, we tested whether the US-CRS score accurately estimates mortality risk for exception candidates, by comparing ROC-AUC for candidates with and without exceptions. We also evaluated whether candidates listed by exception in the current system have similar predicted waitlist mortality to candidates without exceptions, according to the US-CRS score. Specifically, we assessed whether exception requests accurately reflect medical severity as measured by US-CRS, by comparing US-CRS scores among candidates in statuses 1 and 2. We also compared the predicted and observed risk of waitlist mortality among candidates with and exceptions.

## Materials and methods

### Data source

This study used data from the Scientific Registry for Transplant Recipients (SRTR). The SRTR data system includes data on all donor, wait-listed candidates, and transplant recipients in the US, submitted by the members of the Organ Procurement and Transplantation Network (OPTN). The Health Resources and Services Administration (HRSA), US Department of Health and Human Services provides oversight to the activities of the OPTN and SRTR contractors. This dataset has previously been described elsewhere.^10^

### Study population

We included adults aged ≥ 18 years who were added to the heart transplant waiting list between January 1, 2019, and December 31, 2022. To maintain consistency with Zhang et al.,^9^ we reproduced their original center-level data split, in which transplant centers were randomly assigned to training (70%) and testing (30%) sets. For the present study, we used only the original 30% held-out test set from Zhang et al.^9^ (*N* = 4,480). Our analysis was stratified by medical urgency status. Candidates with exceptions were identified using the status justification form dataset as those who did not meet the standard criteria for a given status but were approved by the RRB based on comparable risk. We considered candidates with exceptions as those with an approved exception request that was not an extension.

### US-CRS

The US-CRS uses 7 variables: albumin, log bilirubin, estimated glomerular filtration rate (eGFR), log B-type natriuretic peptide (BNP), N-terminal pro-brain natriuretic peptide (BNP NT Pro), durable left-ventricular assist device (LVAD), and short-term mechanical circulatory device ever used (short MCS ever).^9^ Short-term MCS excluded intra-aortic ballon pump (IABP) and percutaneous endovascular left ventricular assist devices (VAD), and included only extracorporeal membrane oxygenation (ECMO), temporary surgical LVAD, and biventricular assist device without discharge. While the final US-CRS score included only 7 variables, the team that created US-CRS considered and tested 46 potential predictors (43 of which varied over time), including hemodynamics, level of inotropic support, age, diagnosis, diabetes, mechanical circulatory support device (MCS), and MCS complications. The 7 variables in the US-CRS model were selected based on coefficient significance, the area under the receiver operating characteristic curve, value of the Akaike information criterion in the training dataset, and ease of implementation. In Zhang et al.,^9^ sensitivity analyses are available that include/exclude variables such as pulmonary capillary wedge pressure (PCWP), cardiac pressure output (CPO) and aortic pulsatility index (API), cardiac pressure output (CPO), pulmonary artery pulsatility index (PAPI), aspartate aminotransferase (AST) (and different combinations of each), no hemodynamics, and others; none of these changes improved the predictive power of US-CRS.

### Performance of US-CRS in exception candidates versus non-exception candidates

We estimated and plotted the ROC-AUC curves separately for candidates with and without exceptions within high-priority (Status 1 and 2) and lower-priority (Status 3 and 4) groups. We excluded Status 5 and 6 from this analysis because no candidates in these statuses have exceptions. We also assessed model calibration in candidates with and without exceptions by comparing the observed and predicted 6-week waiting list mortality by US-CRS quintiles.

### Waitlist mortality risk as measured by US-CRS

We calculated the US-CRS distribution among candidates with exceptions and candidates without exceptions in Status 1 and 2, and compared their demographic, clinical, laboratory, and device-use characteristics. We also computed the proportion of candidates below specific US-CRS thresholds. For candidates without exceptions, we used their status at initial listing and corresponding test variables to calculate their US-CRS. For candidates with exceptions, we used their first approved exception status and calculated their US-CRS using test variables at the time of their exception request. If a candidate ever had an approved exception request, they were included in the candidates with exceptions group. Candidates who appeared in both groups at different points in time (12 Status 1 candidates and 58 Status 2 candidates) were excluded from the candidates without exceptions group. We conducted a sensitivity analysis by including these candidates in both groups (see Supplement).

### Statistical analysis

We used the Kolmogorov-Smirnov (KS) test to compare the US-CRS distributions and the proportion of candidates below specific thresholds (0–20, 20–30, 30–40, 40+) between groups. A US-CRS below 40 indicates lower predicted risk of waitlist mortality than 20% of patients in the US-CRS modeling cohort and may help identify candidates whose exception requests were approved despite being less critically ill. We compared the demographic, clinical, laboratory, and device-use characteristics of candidates with exceptions to candidates without exceptions using Wilcoxon Rank-Sum test for continuous variables and Pearson’s Chi-squared test or Fisher exact test for categorical variables (p≤0.05). All analyses were performed using R Version 4.0.3 (R Foundation for Statistical Computing, Vienna, Austria).

## Results

### Performance of US-CRS in exception candidates versus non-exception candidates

ROC-AUC estimates were similar between candidates with and without exception especially within lower priority group. Among Status 1 and 2 candidates, the AUC for candidates without exceptions was 0.720 (95% CI 0.629–0.801), compared with 0.682 (95% CI 0.594–0.771) for candidates with exceptions (Figure 1). Among Status 3 and 4 candidates, the AUC was 0.823 (95% CI 0.717–0.913) for candidates without exceptions and 0.839 (95% CI 0.768–0.902) for candidates with exceptions (Figure 2). The confidence intervals for each group overlap, meaning that US-CRS is accurate for measuring mortality risk among exception candidates as it is among non-exception candidates. We also report the differences in observed mortality vs. predicted mortality for each of the quintiles in the exception vs. non-exception group (see Supplement).

**Figure 1:**
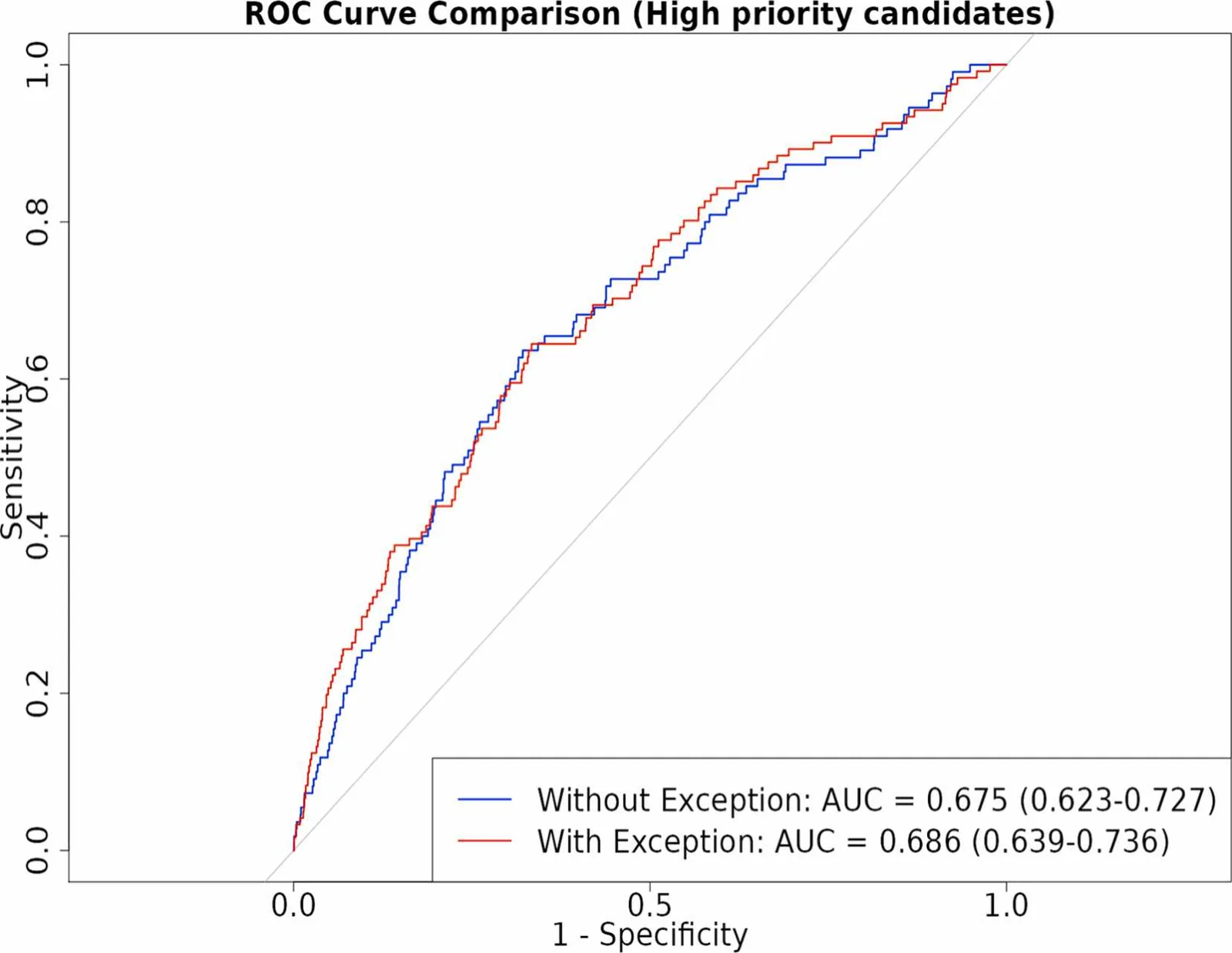
ROC-AUC comparison in high priority status (Status 1 and Status 2).

**Figure 2:**
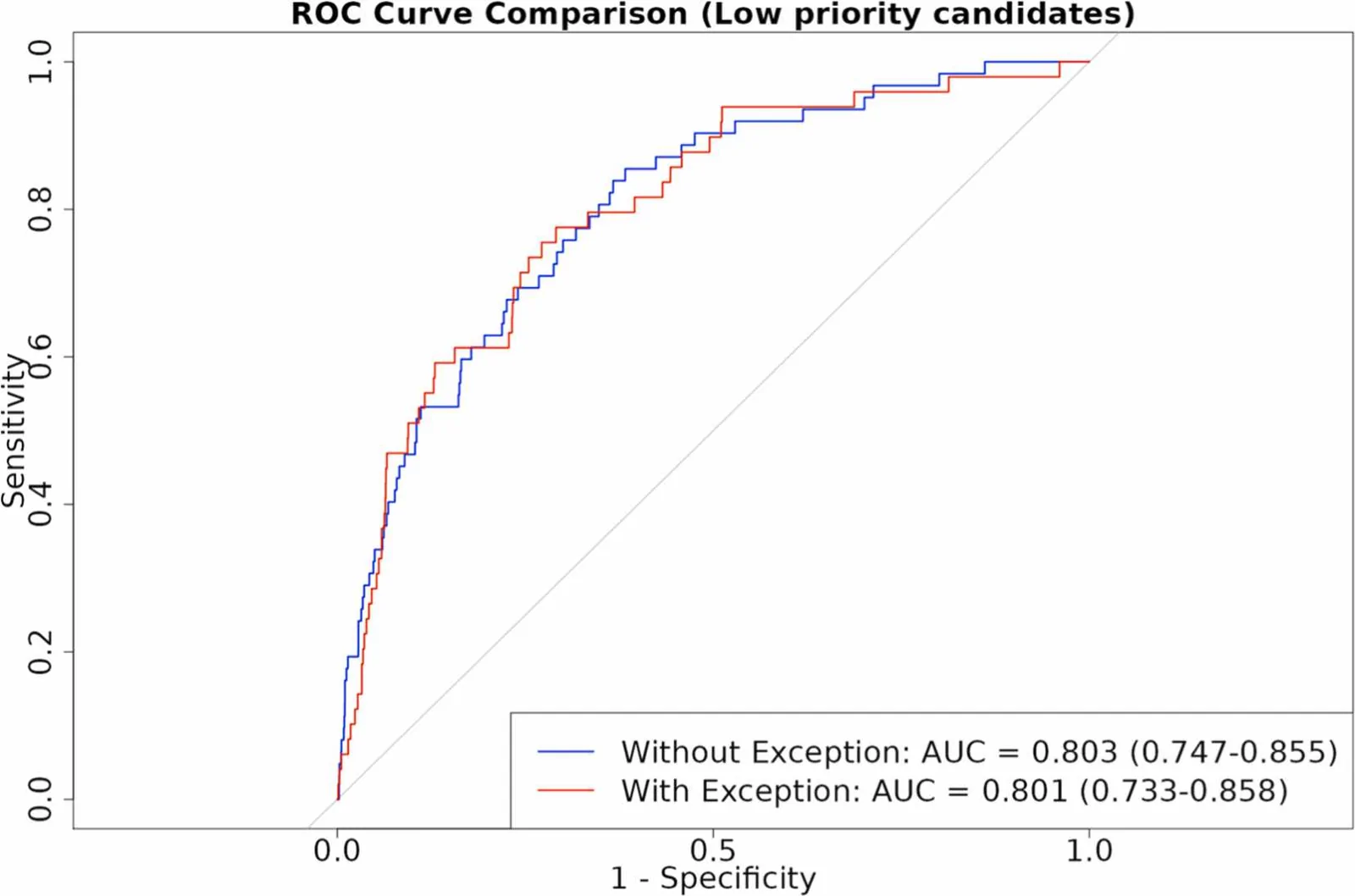
ROC-AUC comparison in low priority status (Status 3 and Status 4).

### Study population

The demographic and clinical characteristics of all Status 1 and 2 candidates, 1,293 with exceptions and 850 without exceptions, appear in Table 1. Among Status 1 candidates with exceptions compared to candidates without exceptions, a substantially higher proportion of candidates with exceptions were Black (34% vs. 25%). However, similar pattern was not found in Status 2.

**Table 1:** Comparison of Population Characteristics, Cardiovascular Measurements, and Cardiac Device Usage in Status 1 and 2 Candidates

	**Status 1 (*N* = 341)**			**Status 2 (*N* = 1,802)**		
	**Without exceptions**^a^**(*N* = 163)**	**With exceptions**^a^**(*N* = 178)**	***p*-value**^b^	**Without exceptions**^a^**(*N* = 687)**	**With exceptions**^a^**(*N* = 1,115)**	***p*-value**^b^
Age (median [IQR])	48 (35, 59)	51 (41, 59)	0.300	**57 (47, 63)**	**55 (42, 62)**	**<0.001**
Sex, *N* (%)						
Female Male	47 (29%) 116 (71%)	45 (25%) 133 (75%)	0.500	142 (21%) 545 (79%)	252 (23%) 863 (77%)	0.300
Race and ethnicity, *N* (%)						
American Indian or Alaska Native Asian Black or AA Hispanic or Latino Multi-racial Native Hawaiian or Other PI White	0 (0%) 0 (0%) 7 (4.3%) 41 (25%) 23 (14%) 2 (1.2%) 90 (55%)	3 (0.6%) 26 (4.8%) 9 (5.1%) 60 (34%) 13 (7.3%) 1 (0.6%) 96 (53%)	0.200	1 (0.1%) 29 (4.2%) 224 (33%) 74 (11%) 5 (0.7%) 1 (0.1%) 353 (51%)	1 (<0.1%) 43 (3.9%) 356 (32%) 118 (11%) 2 (0.2%) 1 (<0.1%) 594 (53%)	0.600
Albumin (median [IQR], g/dl)	**3.10 (2.60, 3.40)**	**3.50 (3.00, 3.90)**	**<0.001**	**3.50 (3.20, 3.80)**	**3.70 (3.20, 4.00)**	**<0.001**
Bilirubin (median [IQR], mg/dl)	**1.40 (0.90, 2.50)**	**0.85 (0.60, 1.40)**	**<0.001**	**1.00 (0.70, 1.60)**	**0.90 (0.60, 1.40)**	**<0.001**
eGFR (median [IQR], ml/min/1.73 m^2^)	**64 (35, 100)**	**47 (29, 78)**	**0.004**	55 (39, 73)	53 (37,74)	0.300
Sodium (median [IQR], mEq/liter)	**137 (133, 140)**	**135 (133, 138)**	**0.011**	**135 (132, 137)**	**136 (133, 138)**	**<0.001**
BNP (median [IQR], pg/ml)	879 (290, 2,965)	1,085 (290, 3,175)	0.600	**1,421 (335, 3,513)**	**923 (290, 2,950)**	**<0.001**
NT pro-BNP, *N* (%)			**0.007**			**<0.001**
0 1	**122 (75%)** **41 (25%)**	**109 (61%)** **69 (39%)**		**433 (63%)** **254 (37%)**	**596 (53%)** **519 (47%)**	
*Cardiovascular measurements*						
Cardiac output (median, [IQR], liter/min)	**3.60 (2.92, 4.50)**	**4.00 (3.30, 5.08)**	**0.002**	**3.87 (3.19, 4.92)**	**4.17 (3.34, 5.20)**	**<0.001**
PAP (median [IQR], mm Hg)						
Systolic	38 (31, 48)	40 (34, 52)	0.120	**45 (38, 55)**	**44 (34, 53)**	**<0.001**
Diastolic	20 (18, 28)	21 (17, 27)	0.500	**23 (18, 29)**	**20 (16, 27)**	**<0.001**
Systolic BP (median [IQR], mm Hg)	**93 (80, 108)**	**103 (92, 110)**	**<0.001**	**92 (85, 106)**	**101 (95, 113)**	**<0.001**
Diastolic BP (median [IQR], mm Hg)	**70 (61, 80)**	**71 (63, 80)**	**0.045**	**67 (59, 79)**	**72 (61, 80)**	**<0.001**
Central venous pressure (median [IQR], mm Hg)	12 (8, 17)	12 (7, 16)	0.400	10 (6, 15)	10 (6, 14)	0.700
Cardiac Power output (median [IQR], W)	**0.57 (0.44, 0.71)**	**0.69 (0.52, 0.88)**	**<0.001**	**0.61 (0.48, 0.80)**	**0.72 (0.56, 0.92)**	**<0.001**
PAPi (median [IQR])	**1.43 (1.00, 1.88)**	**1.67 (1.18, 3.22)**	**<0.001**	2.19 (1.38, 4.00)	2.25 (1.50, 3.83)	0.600
*Cardiac devices*						
ECMO (%)	**72%**	**5.10%**	**<0.001**	0%	0.1%	0.900
Temporary surgical LVAD (%)	0%	0.60%	0.300	**1.9%**	**0.40%**	**0.003**
BiVAD with no discharge (%)	**20%**	**0.00%**	**<0.001**	0%	0.10%	0.900
IABP (%)	**0%**	**5.10%**	**0.004**	**66%**	**3.5%**	**<0.001**
Durable LVAD (%)	**0.6%**	**16.00%**	**<0.001**	**4.8%**	**11%**	**<0.001**
Percutaneous VAD %	**0%**	**4.50%**	**0.006**	**21%**	**1.7%**	**<0.001**
*US-CRS score (median [IQR])*	**50 (48, 50)**	**45 (36, 48)**	**<0.001**	**46 (41, 49)**	**42 (32, 47)**	**<0.001**
<20	**0.00%**	**10.67%**		**3.49%**	**10.76%**	
20–30	**0.00%**	**8.42%**		**2.76%**	**9.99%**	
30–40	**1.84%**	**14.04%**		**12.95%**	**20.53%**	
40+	**98.16%**	**66.85%**		**80.78%**	**58.74%**	

ECMO, Extracorporeal membrane oxygenation; LVAD, Left-ventricular assist device; US-CRS, US-candidate risk score; VAD, Ventricular assist device.

Bold values denote statistically significant differences between candidates with and without exceptions, separately within each status.

a Median (IQR); *n* (%).

b Wilcoxon rank sum test; Pearson’s Chi-Squared test; Fisher’s exact test.

Status 1 candidates without exceptions had higher bilirubin levels (1.4 g/dl vs. 0.8 g/dl; p<0.001) and lower albumin levels (3.1 g/dl vs. 3.5 g/dl; p<0.001) than Status 1 candidates with exceptions (Table 1). Status 1 candidates without exceptions were less likely to have durable LVADs than Status 1 candidates with exceptions (0.6% vs. 16%, p<0.001). Status 1 candidates with exceptions had significantly higher cardiovascular parameters- including cardiac output, cardiac power output, systolic and diastolic blood pressure (BP), than Status 1 candidates without exceptions (all p<0.05).

Status 2 candidates without exceptions had higher bilirubin levels (1.0 vs. 0.9; p<0.001)and lower albumin levels (3.5 vs. 3.7; p<0.001) than Status 2 candidates with exceptions (Table 1). Status 2 candidates without exceptions had lower BP (both systolic and diastolic) and cardiac power output than Status 2 candidates with exceptions (all p<0.001). Status 2 candidates without exceptions had higher use of IABP (p<0.001) than Status 2 candidates with exceptions. Status 2 candidates with exceptions had more durable LVADs (11% vs. 4.8%, p<0.001). Status 2 candidates with exceptions had significantly higher cardiovascular parameters- including cardiac output, cardiac power output, systolic and diastolic BP, than Status 2 candidates without exceptions (all p<0.001).

### Waitlist mortality risk as measured by US-CRS

US-CRS was correlated with priority status (higher US-CRS, higher status) for candidates in Status 1 through 4, but US-CRS distributions in each status differed depending on whether candidates had exceptions (Figure 3). For Status 1, candidates without exceptions had a median [IQR] US-CRS of 50 [48,50] compared to 45 [36,48] for candidates with exceptions (p<0.05) (Figure 4). For Status 2, the median [IQR] US-CRS was 46 [41,49] for candidates without exceptions compared to 42 [32,47] for candidates with exceptions (p<0.05) (Figure 5). Conversely, among candidates in status 3 and 4, candidates with exceptions generally had higher US-CRS than candidates without exceptions (Figure 6, Figure 7).

**Figure 3:**
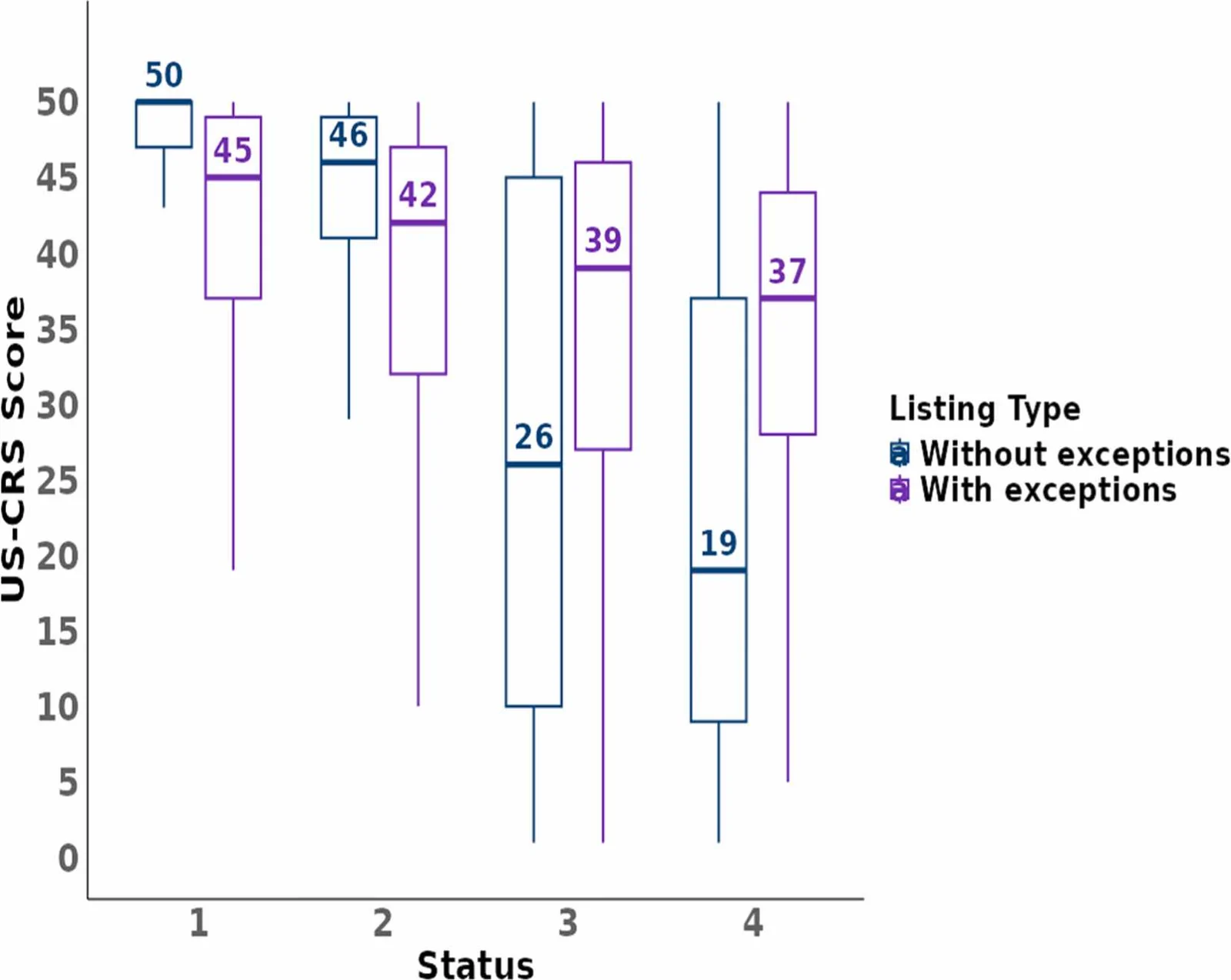
Variation in US-CRS among candidates without exceptions and candidates with exceptions in status 1–4. US-CRS, US-candidate risk score.

**Figure 4:**
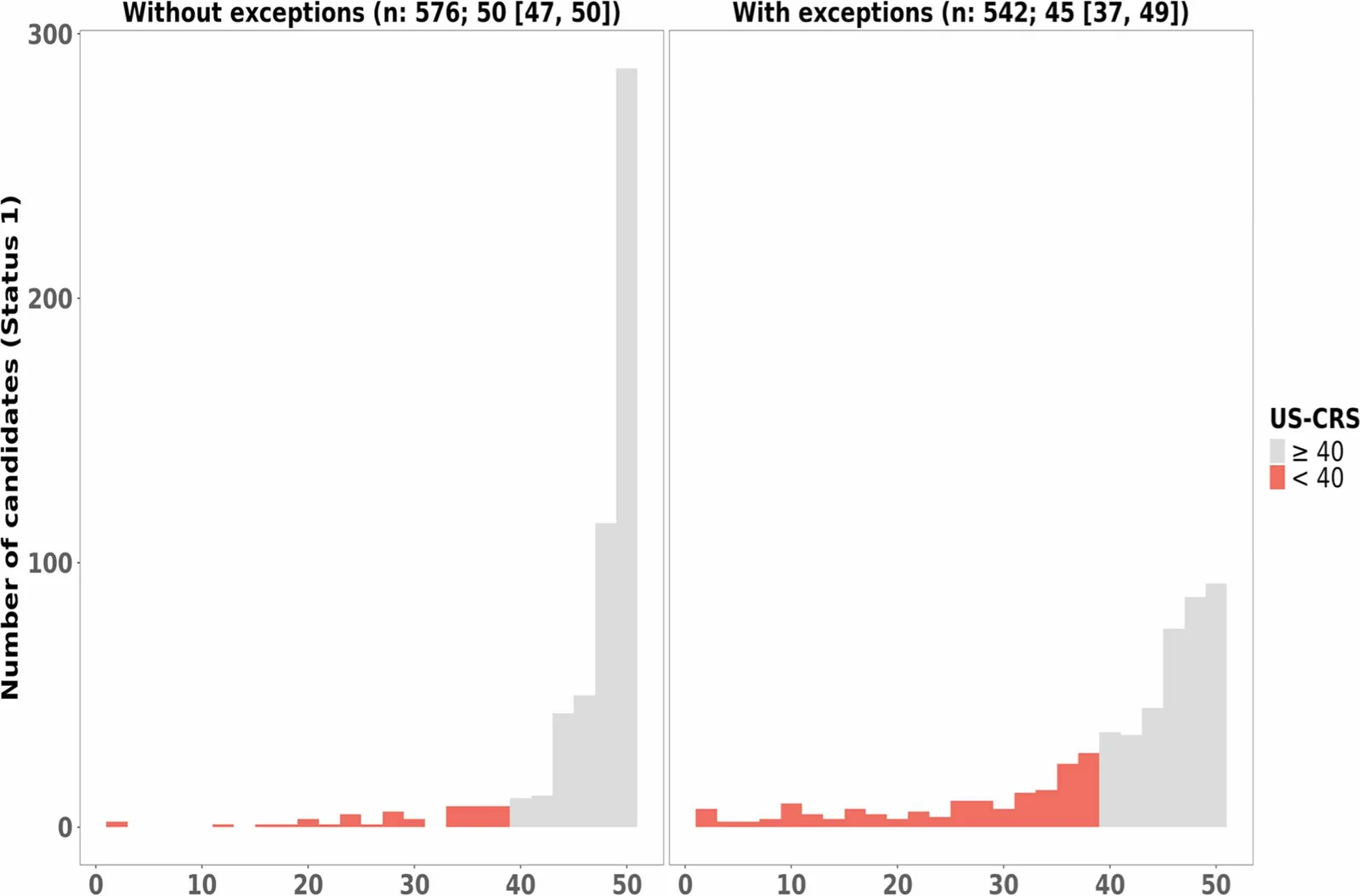
Variation in US-CRS distribution among status 1 candidates. US-CRS, US-candidate risk score.

**Figure 5:**
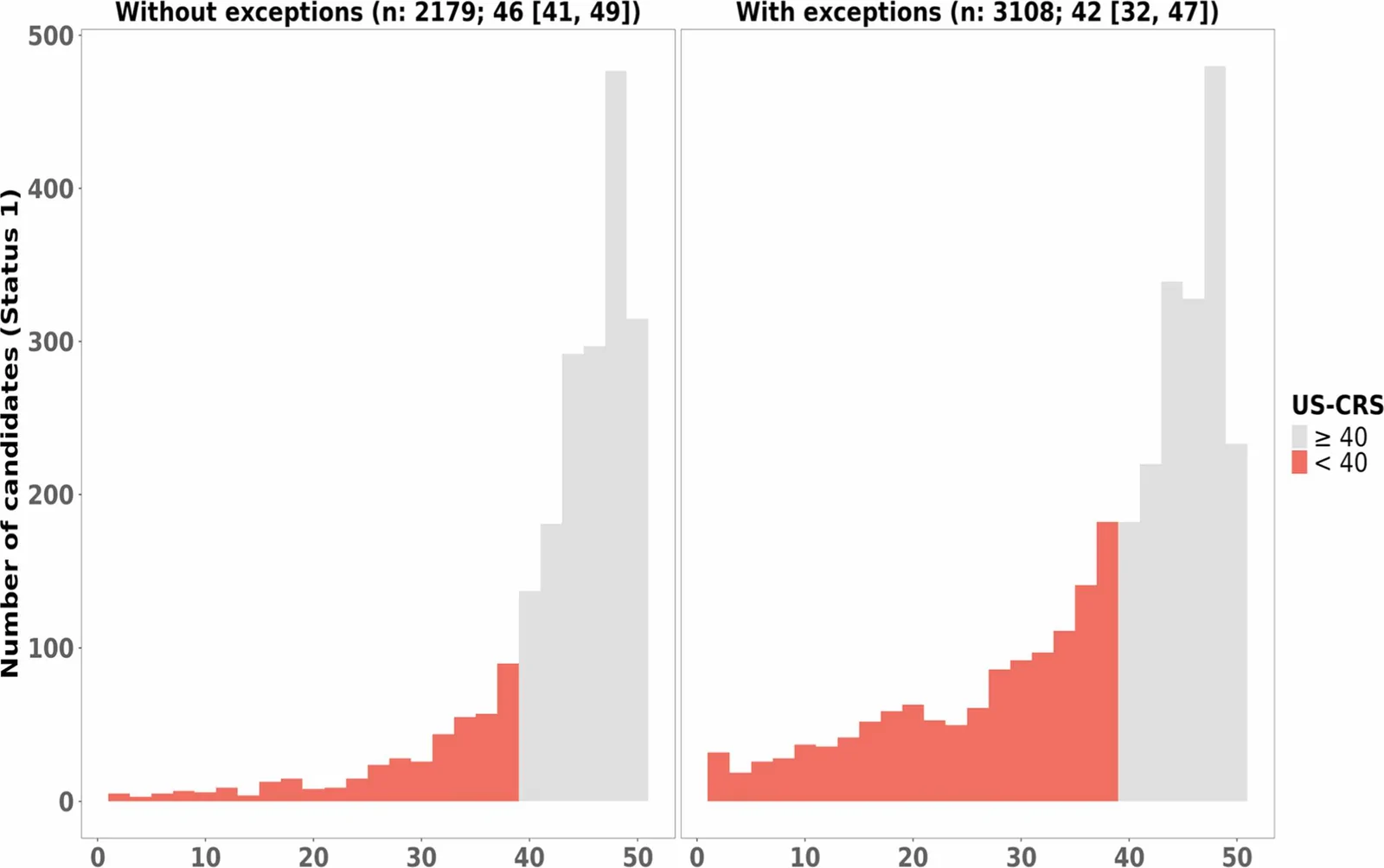
Variation in US-CRS distribution among status 2 candidates. US-CRS, US-candidate risk score.

**Figure 6:**
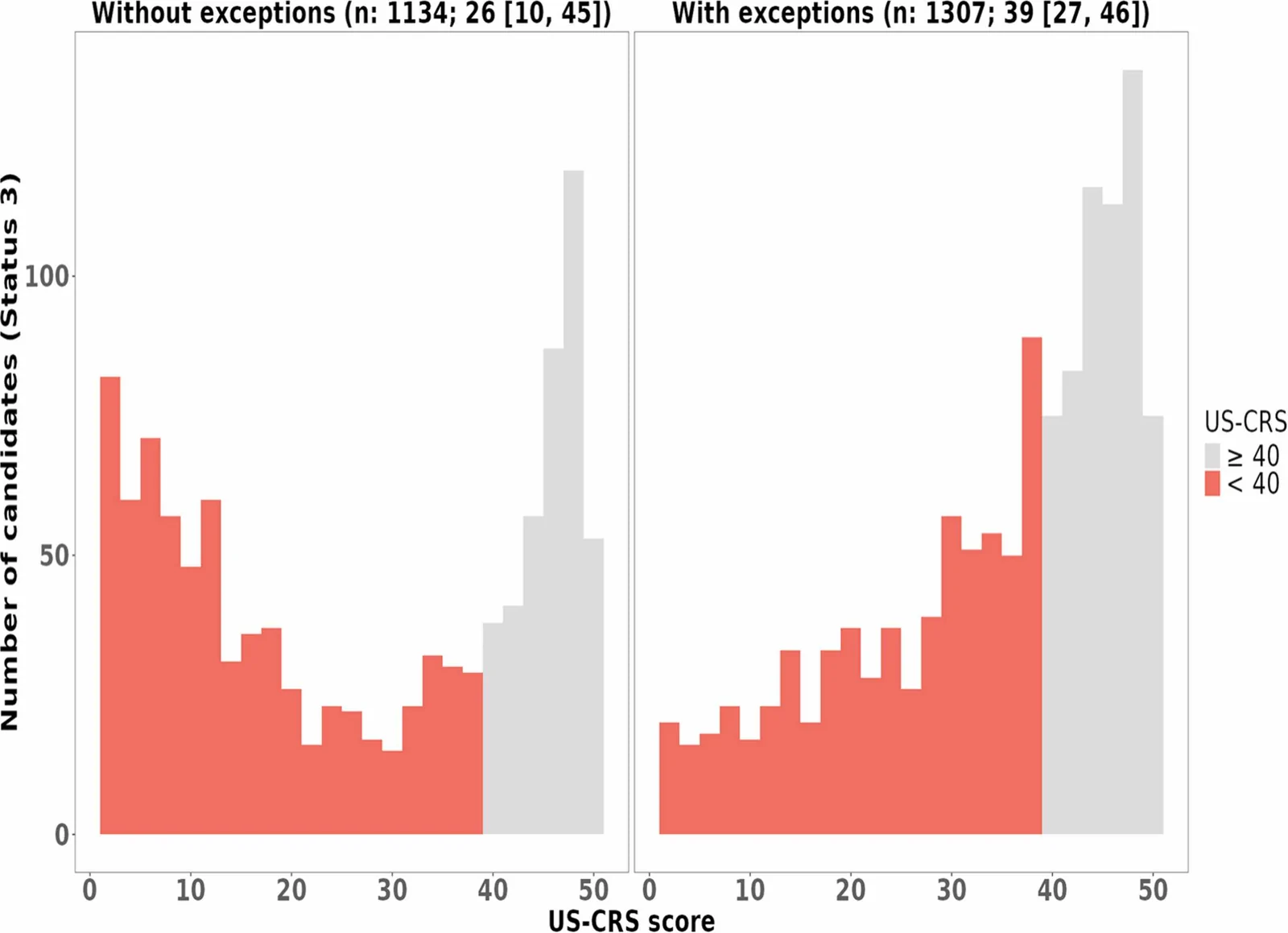
Variation in US-CRS distribution among status 3 candidates. US-CRS, US-candidate risk score.

**Figure 7:**
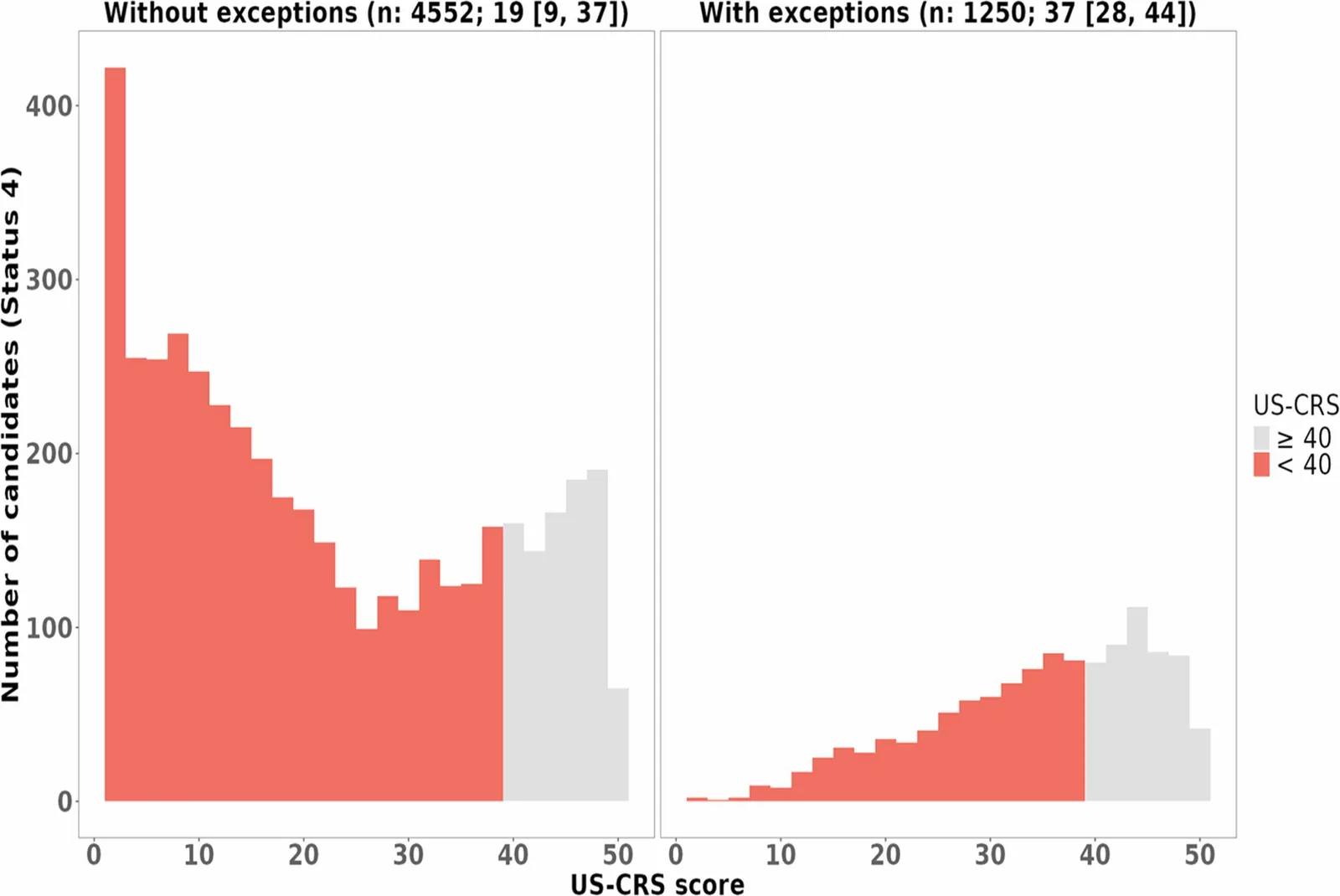
Variation in US-CRS distribution among status 4 candidates. US-CRS, US-candidate risk score.

Notably, 33.13% of Status 1 candidates with exceptions had a US-CRS below 40, compared to only 1.84% of candidates without exceptions. For Status 2, 41.28% of candidates with exceptions had a US-CRS below 40 compared to only 19.20% in without exceptions group, suggesting some candidates with exceptions had lower urgency than candidates without exceptions. Finally, a greater proportion of candidates with exceptions had US-CRS below 20 compared to candidates without exceptions in both Status 1 (10.67% vs. 0.00%) and Status 2 (10.76% vs. 3.49%). We present a schematic showing all candidate statuses and the comparisons performed (Figure 8).

**Figure 8:**
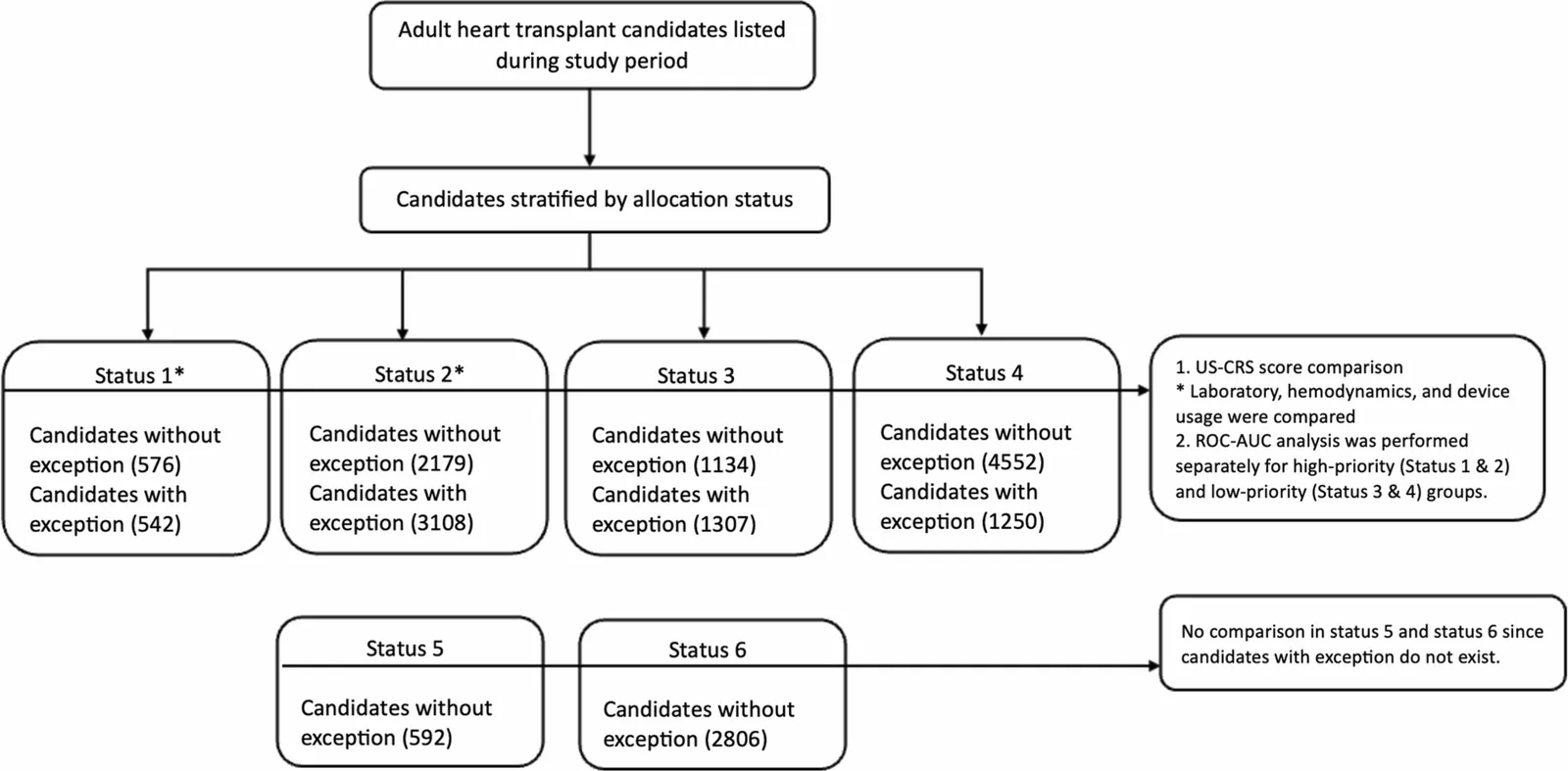
Schematic of candidate status categories and analytical comparisons.

## Discussion

In this registry-based study of US heart transplant candidates, we determined the medical urgency of candidates with exceptions in comparison to candidates without exceptions at higher statuses, as measured by the US-CRS. We found: (1) some alignment between US-CRS and status levels (2) significant differences in distribution of US-CRS between candidates with exceptions and candidates without exceptions, and (3) racial, clinical, hemodynamic, and device usage differences between candidates with and without exceptions at the same status. 33.13% of Status 1 and 41.28% of Status 2 candidates with exceptions had US-CRS scores below 40, compared to only 1.84% and 19.20% of candidates without exceptions, respectively. The US-CRS score is only an imperfect prediction of mortality risk, but it is not a worse predictor of mortality among exception candidates than among non-exception candidates. We evaluated the performance of US-CRS score in estimating mortality risk, and found the performance to be similar for exception candidates and non-exception candidates. We also observed a large proportion of exception candidates in Status 1 (approximately 70%) and in Status 2 (approximately 60%) had US-CRS scores above 40, highlighting the appropriateness of exception requests for such candidates by their physicians.

Multivariable waitlist mortality risk scores have been used in the lung and liver transplant allocation systems to avoid grouping patients with different risk into the same category of transplant priority.^11^, ^12^, ^13^ Despite the 2018 revision expanding the heart allocation system to 6 statuses, heart allocation remains vulnerable to inconsistencies due to widespread usage of exception requests, and due to the imprecision of classifying patients into a few statuses that group patients with heterogeneous mortality risk. Incorporating a risk score like the US-CRS could better identify the sickest candidates.^9^

A significant proportion of candidates with exceptions (33.13%: Status 1; 41.28%: Status 2) had US-CRS scores below 40, suggesting they might have lower mortality risk than their status implied. Prior studies also reported a lower waitlist mortality rate among candidates with exceptions compared to candidates without exceptions in higher statuses.^7^, ^8^ We did not review individual exception narratives, though that might be an area for future research. Physicians submitting exception requests attest that candidates with exceptions have comparable mortality risk to candidates without exceptions in the same status. We also found that a substantial proportion of these candidates in Statuses 1 and 2 had lower US-CRS.^3^, ^14^ Compared to candidates without exceptions, candidates with exceptions also had higher albumin, lower bilirubin, and more favorable hemodynamic parameters, including higher cardiac output, cardiac power output, and blood pressure. However, these hemodynamic differences should be interpreted cautiously given the substantial differences in mechanical circulatory support between groups, as device support may influence both the measured hemodynamics and their clinical interpretation. Notably, hemodynamic variables were evaluated during development of the US-CRS but did not improve model performance and were therefore not included in the final score.

The OPTN Heart committee has expressed concern about the high volume of exception requests and whether they are appropriate, especially for the large number of Status 2 exception requests. In February 2020 the OPTN issued guidance for adult heart exceptions for status 2 candidates that standardized exception request forms to assist review boards in consistently applying policy. The changes include clarifying the type and level of detail required in Status 2 exception requests, particularly for those supported with temporary mechanical circulatory support (MCS) or IABP devices as a bridge to transplant (BTT).^15^ One potential alternative is to incorporate the US-CRS score into existing guidelines for exception request submission, which may help reduce the overall number of exception requests by identifying candidates at lower risk of waitlist mortality.

Some may deem it clinically reasonable, under the current allocation system, to request exceptions for candidates because those candidates are biologically disadvantaged for receiving a compatible offer, and indeed the exception group is enriched for O blood type candidates.^2^ Although candidates with O blood type are prioritized in allocation, that priority is still insufficient; O candidates have lower transplant rates (subhazard ratio: 0.56) and higher rates of waitlist death or deterioration.^16^ Because undersized heart transplants have poor outcomes, the largest candidates are likely at an analogous disadvantage if only a fraction of the offered hearts are large enough.^17^, ^18^ Immunologically sensitized candidates are also likely disadvantaged. Finally, many are frustrated by the low prioritization in the current system for candidates bridged to transplant with LVADs, but most candidates with LVADs have low waitlist mortality risk.^19^, ^20^, ^21^ In all of these instances, the continuous distribution paradigm could grant priority points for patients who need them to achieve equitable access to transplant, separately from their status or US-CRS. Continuous distribution framework envisions a composite allocation score that includes medical urgency (which might or might not be measured with the US-CRS score), post-transplant survival, candidate access and geography, candidate biological disadvantage, and/or other factors; this framework might give alternate weight to some factors that presently lead to heavy use of exceptions. Both the status system and US-CRS are intended to measure waitlist mortality risk, but allocation systems should additionally (and separately) prioritize equity in access to transplant. Biological disadvantage (size, O blood type, sensitization), conditions for which mortality prediction systems are inaccurate (RV dysfunction, congenital heart disease, electrical instability, hypertrophic or restrictive cardiomyopathy), and other patient access factors (pediatric, prior living donors) should be separately prioritized in a future heart allocation system. Patients who are clinically eligible for short-term MCS but unable to receive it (due to contraindications such as significant aortic insufficiency) might have elevated waitlist mortality risk not captured by US-CRS, and could instead be given additional allocation points.

The US-CRS model is similarly calibrated for predicting mortality risk of candidates with exceptions as for patients without exceptions, so the US-CRS might greatly reduce the number of exceptions needed compared with the 6-status system. Exception review boards should consider the US-CRS value for predicted waitlist mortality when reviewing applications, not to determine whether an exception is approved, but to provide reviewers an additional standardized estimate of mortality risk that might identify cases in which the requested priority and estimated urgency are substantially discordant. Our study findings support moving toward a more continuous measure of medical urgency. Adding more categorical tiers might improve risk discrimination incrementally, but the wide risk distributions within statuses suggest that a continuous-risk framework would probably better order candidates by urgency. Exceptions should still be obtainable for clinical circumstances or access disadvantages that are not adequately captured by the risk model. In the near-term, adding categories to separate standard-criteria from exception candidates within a status might improve overall sorting by medical urgency; for example, in decreasing order of priority: status 1 by criteria candidates, then status 1 by exception candidates, then status 2 by criteria candidates, then status 2 by exception candidates.

We acknowledge study limitations. In a very small number of cases, exception requests were initially approved and later disapproved; we do not expect these few instances to meaningfully affect our findings. We did not evaluate whether the US-CRS accurately reflects mortality risk for smaller candidate subgroups, such as multi-organ recipients, candidates clinically ineligible for short-term MCS but unable to receive it, and those with restrictive or hypertrophic cardiomyopathy. Transplant centers should always have a mechanism to request exceptions for mortality risk scores they believe misrepresent their patient’s mortality risk. Evaluating the US-CRS accuracy for these groups is beyond the scope of our study, but we believe these stratified analyses should be performed. Changes in temporary MCS utilization and listing practices since the US-CRS development period may affect its contemporary performance; however, the extent to which such changes would influence relative comparisons between exception and non-exception candidates is uncertain. Our methodology does not directly show that candidates with exception have lower average mortality than without candidates without exception, however prior studies have reported a lower waitlist mortality rate among candidates with exceptions compared to candidates without exceptions in higher statuses. After controlling for status, exception candidates were associated with a 51% lower risk of waitlist mortality among Status 1 candidates (HR 0.49, 95% CI[0.27, 0.91], p = 0.023) and a 61% lower risk among Status 2 candidates (HR 0.39, 95% CI [0.24, 0.62], p < 0.001).^7^ Within these 2 highest priority statuses, exception listings showed consistently lower mortality rates than their standard criteria counterparts (Status 1 HR 0.72, p = 0.028; Status 2 HR 0.76, p = 0.03).^8^

Among Status 3 and 4 candidates, candidates with exceptions generally had higher US-CRS than candidates without exceptions (Status 3: US-CRS: (38 [24,45] vs. (26 [11,45]); Status 4: (19 [9,37] vs. 36 [27,44])). These findings support the continued importance of the exception pathway. Some high-risk candidates remain in the lower status tiers whose standard criteria do not adequately capture their urgency, and exceptions allow them to move toward a priority more consistent with their risk. Thus, the issue is not simply overuse of exception requests, but also the broader problem is that the categorical status system itself does a fairly imperfect job of separating patients by mortality risk, particularly below the very highest-priority groups. While mortality risk as measured by US-CRS is correlated with status, the distribution of US-CRS in candidates with exceptions was lower than for candidates without exceptions, suggesting that exceptions have been granted to candidates with comparatively lower medical urgency. Our findings underscore the need for improved discrimination of waitlist mortality risk among heart transplant candidates, and for considering biological disadvantage and patient access factors in the heart allocation system. Allocation priority under the 6-status system is misaligned with waitlist mortality risk as measured by US-CRS.

## Declaration of Competing Interest

The authors declare that they have no known competing financial interests or personal relationships that could have appeared to influence the work reported in this paper.

## Data Availability

The data reported here have been supplied by the Hennepin Healthcare Research Institute (HHRI) as the contractor for the Scientific Registry of Transplant Recipients (SRTR). The interpretation and reporting of these data are the responsibility of the author(s) and in no way should be seen as an official policy of or interpretation by the SRTR or the US Government.

## Footnotes

Appendix A Supplementary data associated with this article can be found in the online version at doi:10.1016/j.jhlto.2026.100706.
