# Supplementary material for: Waitlist mortality risk (US-CRS score) distribution among status 1 and 2 adult heart transplant candidates with exceptions
Source: JHLT Open. 2026 Oct 12;14:100706. doi: 10.1016/j.jhlto.2026.100706 (PMC13744348; doi:10.1016/j.jhlto.2026.100706)
Supplement: Supplementary file 1 [file mmc1.docx]

**Supporting Information Statement**

**Section S1**. Sensitivity analysis for candidates who had similar status both with and without exceptions.

**Supplemental Figures**

**Figure S1**. Variation in US-CRS score among candidates without exceptions and candidates with exceptions in status 1- 4.

**Figure S2**: Variation in US-CRS score distribution among status 1 candidates.

**Figure S3**: Variation in US-CRS score distribution among status 2 candidates.

**Supplemental Table**

**Table S1:** Comparison of population characteristics, cardiovascular measurements, and cardiac device usage in Status 1 and 2 candidates.

**Table S2**: Predicted vs. observed mortality in exception and non-exception candidates.

**S1. Criteria for inclusion of candidates in the groups of candidates with and without exceptions**

For Status 1, 12 candidates appeared in both the group of candidates with exceptions and without exceptions. For Status 2, 58 candidates appeared in both groups. These overlapping candidate IDs were included in both groups for analysis in this supplement.

**Figure S1**

**Figure S2**

**Figure S3**

**Table S1:** Comparison of population characteristics, cardiovascular measurements, and cardiac device usage in Status 1 and 2 candidates.

|  | **Status 1 (N=353)** | |  | **Status 2 (N=1,860)** | |  |
| --- | --- | --- | --- | --- | --- | --- |
|  | **Without**  **exceptions ^^^ (N=175)** | **With**  **exceptions ^^^**  **(N=178)** | **p-value^*^** | **Without**  **exceptions^^^ (N=745)** | **With**  **exceptions ^^^ (N=1,115)** | **p-value^*^** |
| Age (median (IQR)) | 48 (35, 59) | 51 (41, 59) | 0.200 | 57 (47, 63) | 55 (42, 62) | **<0.001** |
| Sex, N (%)  Female  Male | 52 (30%)  123 (70%) | 45 (25%)  133 (75%) | 0.400 | 150 (20%)  595 (80%) | 252 (23%)  863 (77%) | 0.200 |
| Race and ethnicity, N (%)  American Indian or Alaska Native  Asian  Black or AA  Hispanic or Latino  Multi-racial  Native Hawaiian or Other PI  White | 0 (0%)  0 (0%)  9 (5.1%)  44 (25%)  23 (13%)  2 (1.1%)  97 (55%) | 0 (0%)  0 (0%)  9 (5.1%)  60 (34%)  13 (7.3%)  1 (0.6%)  95 (53%) | 0.2 | 1 (0.1%)  29 (3.9%)  244 (33%)  83 (11%)  6 (0.8%)  2 (0.3%)  380 (51%) | 1 (<0.1%)  43 (3.9%)  356 (32%)  118 (11%)  2 (0.2%)  1 (<0.1%)  594 (53%) | 0.400 |
| Albumin (median (IQR), g/dL) | **3.10 (2.60, 3.40)** | **3.50 (3.00, 3.90)** | **<0.001** | **3.50 (3.20, 3.80)** | **3.70 (3.20, 4.00)** | **<0.001** |
| Bilirubin (median (IQR), mg/dL) | **1.30 (0.80, 2.40)** | **0.85 (0.60, 1.40)** | **<0.001** | **1.00 (0.70, 1.60)** | **0.90 (0.60, 1.40)** | **<0.001** |
| eGFR (median (IQR), mL/min/1.73 m^2^) | **64 (35, 100)** | **47 (29, 78)** | **0.005** | 55 (39, 73) | 53 (37,74) | 0.300 |
| Sodium (median (IQR), mEq/L) | **137 (133, 140)** | **135 (133, 138)** | **0.007** | **135 (132, 137)** | **136 (133, 138)** | **<0.001** |
| BNP (median (IQR), pg/mL) | 968 (290, 3,277) | 1,058 (290, 3,175) | >0.900 | **1,393 (335, 3,513)** | **923 (290, 2,950)** | **<0.001** |
| NT pro-BNP, N (%)  0  1 | **128 (73%)**  **47 (27%)** | **109 (61%)**  **69 (39%)** | **0.017** | **457 (63%)**  **278 (37%)** | **596 (53%)**  **519 (47%)** | **<0.001** |
| **Cardiovascular measurements** | | | | | |  |
| Cardiac output (median, (IQR), L/min) | **3.60 (2.95, 4.52)** | **4.00 (3.30, 5.08)** | **0.005** | **3.90 (3.20, 4.97)** | **4.17 (3.34, 5.20)** | **<0.001** |
| PAP (median (IQR), mm Hg) |  |  |  |  |  |  |
| Systolic | 38 (32, 47) | 40 (34, 52) | 0.079 | **45 (38, 55)** | **44 (34, 53)** | **<0.001** |
| Diastolic | 20 (18, 28) | 21 (17, 27) | 0.600 | **23 (18, 29)** | **20 (16, 27)** | **<0.001** |
| Systolic BP (median (IQR), mm Hg) | **94 (81, 108)** | **103 (92, 110)** | **<0.001** | **92 (85, 105)** | **107 (95, 113)** | **<0.001** |
| Diastolic BP (median (IQR), mm Hg) | 70 (62, 80) | 71 (63, 80) | 0.059 | **67 (59, 79)** | **72 (61, 80)** | **<0.001** |
| Central venous pressure (median (IQR), mm Hg) | 12 (8, 17) | 12 (7, 16) | 0.300 | 10 (6, 15) | 10 (6, 14) | 0.800 |
| Cardiac Power output (median (IQR), W) | **0.57 (0.45, 0.72)** | **0.69 (0.52, 0.88)** | **<0.001** | **0.61 (0.48, 0.80)** | **0.72 (0.56, 0.92)** | **<0.001** |
| PAPi (median (IQR)) | **1.43 (1.00, 1.86)** | **1.67 (1.18, 3.22)** | **<0.001** | **2.18 (1.38, 4.00)** | **2.25 (1.50, 3.83)** | **0.500** |
| **Cardiac devices** | | | | | |  |
| ECMO (%) | **73%** | **5.10%** | **<0.001** | 0% | 0.1% | >0.900 |
| Temporary surgical LVAD (%) | 0% | 0.60% | 0.300 | **1.9%** | **0.40%** | **0.003** |
| BiVAD with no discharge (%) | **20%** | **0.00%** | **<0.001** | 0% | 0.10% | >0.900 |
| IABP (%) | **0%** | **5.10%** | **0.003** | **66%** | **3.5%** | **<0.001** |
| Durable LVAD (%) | **1.1%** | **16.00%** | **<0.001** | **4.6%** | **11%** | **<0.001** |
| Percutaneous VAD % | **0%** | **4.50%** | **0.005** | **23%** | **1.7%** | **<0.001** |
| **US-CRS score (median [IQR])** | **50 [48, 50]** | **45 [36, 48]** | **<0.001** | **46 [41, 48]** | **42 [32, 47]** | **<0.001** |
| < 20 | **0.00%** | **10.67%** |  | **3.22%** | **10.76%** |  |
| 20-30 | **0.00%** | **8.42%** |  | **2.95%** | **9.99%** |  |
| 30-40 | **4.00%** | **14.04%** |  | **12.75%** | **20.53%** |  |
| 40+ | **96.00%** | **66.85%** |  | **81.07%** | **58.74%** |  |

*^: Median (IQR); n (%); *: Wilcoxon rank sum test; Pearson’s Chi-Squared test; Fisher’s exact test.*

**Table S2:** Predicted vs. observed mortality in exception and non-exception candidates.

| US-CRS risk stratum | Exception, N | Predicted 6-week mortality | Observed  6-week mortality (95% CI) | Non-exception, N | Predicted 6-week mortality | Observed  6-week mortality (95% CI) |
| --- | --- | --- | --- | --- | --- | --- |
| Quintile 1  (lowest predicted risk) | 185 | 0.002 | 0.002  (0.000, 0.003) | 314 | 0.002 | 0.002  (0.001, 0.003) |
| Quintile 2 | 342 | 0.005 | 0.003  (0.001, 0.005) | 428 | 0.003 | 0.005  (0.003, 0.006) |
| Quintile 3 | 465 | 0.008 | 0.004  (0.002, 0.006) | 503 | 0.004 | 0.004  (0.002, 0.006) |
| Quintile 4 | 611 | 0.013 | 0.020  (0.011, 0.019) | 799 | 0.008 | 0.011  (0.008 0.013) |
| Quantile 5  (highest predicted risk) | 847 | 0.037 | 0.036  (0.030, 0.043) | 1308 | 0.032 | 0.038  (0.034, 0.044) |
